# Risk factors for the development of severe typhoid fever in Vietnam

**DOI:** 10.1186/1471-2334-14-73

**Published:** 2014-02-10

**Authors:** Christopher M Parry, Corinne Thompson, Ha Vinh, Nguyen Tran Chinh, Le Thi Phuong, Vo Anh Ho, Tran Tinh Hien, John Wain, Jeremy J Farrar, Stephen Baker

**Affiliations:** 1 Wellcome Trust Major Overseas Programme, Oxford University Clinical Research Unit, Hospital for Tropical Diseases, 764 Vo Van Kiet, District 5, Ho Chi Minh City, Vietnam; 2 Department of Clinical Sciences, Liverpool School of Tropical Medicine, Pembroke Place, L3 5QA, Liverpool, UK; 3 Nuffield Department of Clinical Medicine, Centre for Tropical Medicine, Churchill Hospital, Oxford, UK; 4 Hospital for Tropical Diseases, 764 Vo Van Kiet, District 5, Ho Chi Minh City, Vietnam; 5 Dong Thap Provincial Hospital, Cao Lanh, Dong Thap Province, Vietnam; 6 Department of Medical Microbiology, University of East Anglia, Norwich, UK; 7 London School of Hygiene and Tropical Medicine, Keppel Street, WC1E 7HT, London, UK

**Keywords:** *Salmonella enterica* serovar Typhi, Severe typhoid, Antimicrobial resistance, Multidrug resistance, Intermediate ciprofloxacin susceptibility

## Abstract

**Background:**

Typhoid fever is a systemic infection caused by the bacterium *Salmonella enterica* serovar Typhi. Age, sex, prolonged duration of illness, and infection with an antimicrobial resistant organism have been proposed risk factors for the development of severe disease or fatality in typhoid fever.

**Methods:**

We analysed clinical data from 581 patients consecutively admitted with culture confirmed typhoid fever to two hospitals in Vietnam during two periods in 1993–1995 and 1997–1999. These periods spanned a change in the antimicrobial resistance phenotypes of the infecting organisms i.e. fully susceptible to standard antimicrobials, resistance to chloramphenicol, ampicillin and trimethoprim-sulphamethoxazole (multidrug resistant, MDR), and intermediate susceptibility to ciprofloxacin (nalidixic acid resistant). Age, sex, duration of illness prior to admission, hospital location and the presence of MDR or intermediate ciprofloxacin susceptibility in the infecting organism were examined by logistic regression analysis to identify factors independently associated with severe typhoid at the time of hospital admission.

**Results:**

The prevalence of severe typhoid was 15.5% (90/581) and included: gastrointestinal bleeding (43; 7.4%); hepatitis (29; 5.0%); encephalopathy (16; 2.8%); myocarditis (12; 2.1%); intestinal perforation (6; 1.0%); haemodynamic shock (5; 0.9%), and death (3; 0.5%). Severe disease was more common with increasing age, in those with a longer duration of illness and in patients infected with an organism exhibiting intermediate susceptibility to ciprofloxacin. Notably an MDR phenotype was not associated with severe disease. Severe disease was independently associated with infection with an organism with an intermediate susceptibility to ciprofloxacin (AOR 1.90; 95% CI 1.18-3.07; p = 0.009) and male sex (AOR 1.61 (1.00-2.57; p = 0.035).

**Conclusions:**

In this group of patients hospitalised with typhoid fever infection with an organism with intermediate susceptibility to ciprofloxacin was independently associated with disease severity. During this period many patients were being treated with fluoroquinolones prior to hospital admission. Ciprofloxacin and ofloxacin should be used with caution in patients infected with *S*. Typhi that have intermediate susceptibility to ciprofloxacin.

## Background

Typhoid fever, caused by *Salmonella enterica* serovar Typhi (*S*. Typhi) and *Salmonella enterica* serovar Paratyphi A (*S*. Paratyphi A), has been estimated to cause approximately 27 million infections each year worldwide [1]. The disease is common in parts of Asia particularly among children [2]. Fatality rates in the pre-antimicrobial era ranged from 7 – 26% of hospitalised cases [3-5]. The introduction of chloramphenicol, and subsequently, other antimicrobial agents, led to a marked reduction in the occurrence of severe and fatal disease [4,5]. Despite improvements in treatment, some patients, particularly the very young and elderly, and those receiving inadequate antimicrobial therapy, continue to develop severe and life threatening disease [5-7].

Since the early1990s multidrug-resistant (MDR) strains of *S*. Typhi and *S*. Paratyphi A (resistant to chloramphenicol, trimethoprim/sulphamethoxazole and ampicillin) have not only caused epidemics, but have become endemic across some parts of Asia [2,8]. Similarly, isolates of *S*. Typhi and *S*. Paratyphi A with intermediate susceptibility to ciprofloxacin (formerly called decreased susceptibility and indicated in the laboratory by resistance to nalidixic acid or with a minimum inhibitory concentration (MIC) between 0.1 and 0.5 μg/mL against ciprofloxacin) are now common in endemic areas and in imported infections in industrialised countries [8,9]. Intermediate susceptibility to ciprofloxacin has been associated with longer fever clearance time and treatment failures when either ciprofloxacin or ofloxacin are used for treatment [10,11].

Severe or fatal typhoid fever has been reported to be associated with extremes of age, female and male sex and prolonged illness [4,6,7,12-16]. Infection with antimicrobial resistant isolates (MDR and nalidixic acid resistant) has also been proposed to correlate with the development of severe infection [6,16,17]. The association of antimicrobial resistance with severe disease may result from a delay in the initiation of appropriate antimicrobial therapy, but may also potentially be the result of a genotypic association that increases the pathogenic potential of infecting organism.

We aimed to determine microbiological and clinical factors associated with increased risk of severe typhoid fever, hypothesising that the changing antimicrobial resistance phenotypes throughout the selected study periods in Vietnam were associated with a shift in disease severity. We used a logistic regression model to assess the influence of age, sex, duration of illness prior to hospital admission, and the antimicrobial resistance profile of the infecting isolate on the clinical severity of typhoid.

## Methods

### Patients studied

This analysis was performed on data from patients at two hospitals in southern Vietnam between May 1993-July 1995 and April 1997-February 1999. The two hospitals were The Hospital for Tropical Diseases (HTD) in Ho Chi Minh City (HCMC) and Dong Thap Provincial Hospital (DTPH) in Cao Lanh, Dong Thap Province. HTD is a 500-bed infectious disease hospital with a catchment area encompassing HCMC and is a referral hospital for the surrounding province and DTPH is a 400-bed general hospital, serving the population of Cao Lanh and is a referral centre for the province of Dong Thap in the Mekong Delta. During these time periods consecutive patients with blood or bone marrow culture confirmed typhoid were included in antimicrobial treatment, diagnostic, pathophysiology and surveillance studies at the two hospitals [17-27]. There were only a small number of patients with paratyphoid and they were excluded from this analysis.

Each individual study independently received approval from the scientific and ethical committee of the respective hospital prior to the initiation of the study. Patients, or the parent or guardian for children, gave informed verbal consent before entry into the studies. The studies were conducted in accordance with the Declaration of Helsinki. This was a post-hoc analysis of the existing prospectively collected anonymised patient and laboratory data from these studies. No additional data collection or laboratory analysis was performed.

### Clinical data

Demographic, clinical and laboratory data was prospectively gathered on standardised case report forms at the time of hospital admission. For continuous variables patients were categorised according to the presence within the first 24 hours of admission of; a peak temperature of > 40.0°C, a systolic blood pressure of < 90 mmHg, a haematocrit of < 30%, an elevated white cell count (defined as > 15 × 10^9^/L), a low white cell count (defined as < 4 × 10^9^/L), a low platelet count (defined as < 100 × 10^9^/L), an elevated SGOT (defined as > 200 IU/L which was approximately five times upper limit of normal) and an elevated SGPT (defined as > 200 IU/L which was approximately five times upper limit of normal). Clinical outcomes (death or resolution) were recorded for all patients. Patients who were readmitted as relapse cases were only included for the initial admission. Information concerning antimicrobial agents used for treatment prior to hospital admission was not reliably available.

Severe disease was defined by the presence of one or more of the following features: gastrointestinal bleeding (the presence of visible blood in the stool), intestinal perforation (confirmed at surgery), encephalopathy (delirium, obtundation or coma), haemodynamic shock (systolic blood pressure < 90 mmHg and/or diastolic blood pressure < 60 mmHg associated with tissue hypoperfusion), myocarditis (tachycardia or bradycardia with an associated abnormality of the ECG or ultrasound evidence of a pericardial effusion), hepatitis (as indicated by jaundice and/or hepatomegaly with abnormal levels of SGOT (> 400 IU/L) and/or SGPT (>400 IU/L)), a clinical diagnosis of cholecystitis (right upper quadrant pain and tenderness without evidence of hepatitis), pneumonia (respiratory symptoms with abnormal chest X-ray infiltrates) or pleural effusion, severe anaemia (haematocrit ≤ 20%), the need for a blood transfusion, or death.

### Microbiological methods

Blood or bone marrow aspirates was added to brain heart infusion broth containing sodium polyethanolsulphonate (1:10) and incubated at 35-37°C for seven days. Sub-cultures were performed after one, two and seven days or when the broth went turbid. Alternatively, blood was inoculated into BACTEC bottles (Becton-Dickenson, USA) and cultured for five days in a BACTEC 9050 automated incubator. Bottles that gave a positive signal were sub-cultured. *Salmonella* isolates were identified by standard biochemical tests and agglutination with *Salmonella* specific antisera (Murex diagnostics, Dartford, United Kingdom). Antimicrobial sensitivities were performed at the time of isolation by a modified Bauer-Kirby disc diffusion method and inhibition zone sizes were recorded. Interpretations of the zone sizes were based on the current (2013) CLSI guidelines [28]. The antimicrobials tested (Unipath, Basingstoke, United Kingdom) were chloramphenicol (30μg), ampicillin (10μg), trimethoprim-sulphamethoxazole (1.25/23.75μg), ceftriaxone (30μg), ofloxacin (5μg), azithromycin (15μg) and nalidixic acid (30μg). Isolates were stored in Protect beads (Prolabs, Oxford, United Kingdom) at −20°C.

Stored bacterial isolates were later sub-cultured onto nutrient agar and the MICs for the isolates were determined by the standard agar plate dilution method according to CLSI guidelines or using E- test strips according to the manufacturer’s guidelines (AB Biodisk, Solna, Sweden). The evaluated antimicrobials were ciprofloxacin (0.008 μg/mL to 4 μg/mL), ofloxacin (0.008 μg/mL to 4 μg/mL) and nalidixic acid (0.5μg/mL to 512 μg/mL). Antimicrobial powders were purchased from Sigma, United Kingdom, except for ofloxacin, which was donated from Hoescht Marion Roussel. *Escherichia coli* ATCC® 25922 and *Staphylococcus aureus* ATCC® 25923 were used as control strains for these assays. An isolate was defined as MDR if it was resistant to chloramphenicol (≥ 32μg/ml), trimethoprim/sulphamethoxazole (≥ 8/152 μg/ml) and ampicillin (≥ 32μg/ml). An isolate was defined as having intermediate ciprofloxacin susceptibility if it was resistant to nalidixic acid (≥ 32μg/ml) and/or had a ciprofloxacin MIC of 0.1-0.5 μg/ml and/or ofloxacin MIC of 0.25-1.0 μg/ml.

### Analysis

Demographic and clinical features were described for the whole cohort and within the stratified age categories of < 5 years, 5–15 years and ≥ 16 years. Continuous data was described using median and inter-quartile range and compared using the Mann Whitney *U*-test. Proportions were compared with the Chi squared test or the Fisher’s exact test as appropriate. Age, sex, duration of illness prior to admission to hospital, the presence of an MDR phenotype and intermediate susceptibility to ciprofloxacin were evaluated for association with severe or fatal typhoid fever through a univariate analysis. A multivariate logistic regression model controlling simultaneously for the effects of confounding included variables associated with the outcome of severity (p < 0.10) as well as *a priori* factors age, sex and site. Statistical analysis was performed using STATA version 11 (StataCorp, Texas, USA).

## Results

### Demographic data and diagnostic test results

**Figure 1:**
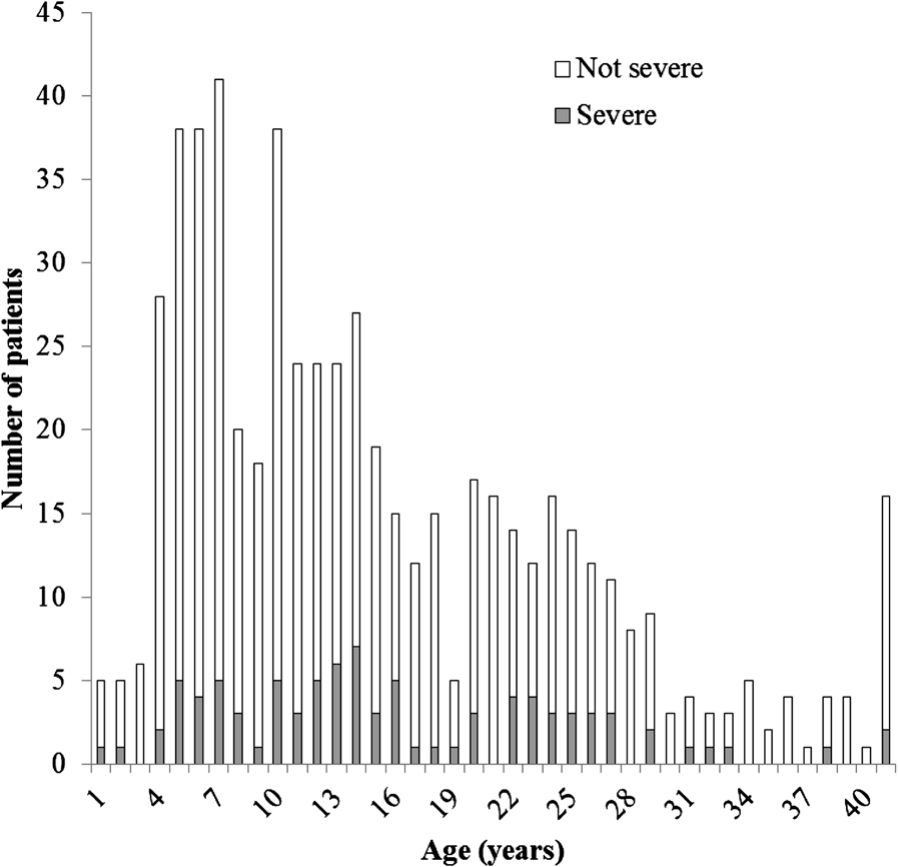
The age distribution and severity in 581 hospitalised Vietnamese patients with typhoid fever between 1993 and 1999.

Data was available for analysis on a total of 581 patients with culture confirmed typhoid fever. There were 347 typhoid patients during 1993 and 1995 and 234 during 1997 and 1999. The final dataset included 355 (61.1%) children (< 16 years), with 44 (7.6%) under the age of 5 years, and 226 (38.9%) adults (≥ 16 years) (Figure 1). There were 296 (50.9%) males, comprised of 196 (55.2%) male children and 100 (43.2%) male adults. A blood and bone marrow culture was performed with material from 193 (33%) patients and blood culture alone was performed on 388 (67%) patients. Notably, in the patients receiving both blood and bone marrow cultures, *S*. Typhi was isolated from both samples on 145 (25%) occasions and from the bone marrow alone on 28 (5%) occasions.

### The clinical and microbiological features of typhoid on hospital admission

The signs and symptoms of the enrolled patients at the time of admission are summarised in Table 1 and classified by age range. Children under 16 years old were more likely than adults to have a shorter duration of illness, constipation and a cough. Abdominal pain and vomiting was most commonly recorded in school-aged children, and adults were more likely than children to have a headache. More than half of all enrolled subjects had diarrhoea and a peak temperature in excess of 40°C. Children under 16 years old were more likely to have hepatopslenomegaly and were more frequently infected with an MDR or intermediate ciprofloxacin susceptibility organism.

**Table 1:** The demographics and clinical features of 581 typhoid fever patients categorised by age

**Covariate**	**All ages**	**<5 years**	**5**-**15 years**	**≥16 years**	**p value**^**1**^	**p value**^**2**^
	**n = 581**	**n = 44**	**n = 311**	**n = 226**		
**Baseline**						
Year^3^						
1993-1995	347 (59.7)	26 (7.5)	177 (51.0)	144 (41.5)	0.785	0.117
1997-1999	234 (40.3)	18 (17.7)	134 (57.3)	82 (35.0)		
Site^4^						
Ho Chi Minh City	350 (60.2)	24 (6.9)	136 (38.9)	190 (54.3)	0.177	<0.001
Dong Thap	231 (39.8)	20 (8.7)	175 (75.6)	36 (15.6)		
Male sex	296 (50.9)	28 (63.6)	168 (53.8)	100 (45.3)	0.230	0.010
Days ill prior to admission^5^	8 (6–11)	7 (6–10)	7 (6–10)	9 (7–14)	0.813	<0.001
**Signs & symptoms**						
Abdominal pain	178 (30.6)	9 (20.5)	129 (41.5)	40 (17.7)	0.007	<0.001
Constipation	134 (23.1)	10 (22.7)	107 (34.4)	17 (7.5)	0.123	<0.001
Diarrhoea	321 (55.2)	26 (59.1)	177 (56.9)	118 (52.2)	0.785	0.240
Vomiting	116 (20)	5 (11.4)	75 (24.1)	36 (15.9)	0.081	0.052
Cough	131 (22.5)	15 (34.1)	79 (25.4)	37 (16.4)	0.221	0.004
Headache	220 (37.9)	5 (11.4)	117 (37.6)	98 (43.4)	<0.001	0.029
Temperature >40°C	318 (54.7)	27 (61.4)	180 (58.6)	111 (53.1)	0.730	0.175
^6^SBP <90 mmHg	25 (4.3)	2 (4.5)	14 (4.5)	9 (4.0)	1.000	0.761
Hepatomegaly	303 (52.2)	31 (70.5)	222 (71.4)	50 (22.1)	0.899	<0.001
Jaundice	17 (2.9)	0 (0)	11 (3.5)	6 (2.7)	0.372	0.757
Splenomegaly	101 (17.4)	10 (22.7)	72 (23.2)	19 (8.4)	0.950	<0.001
Rash	15 (2.6)	1 (2.3)	10 (3.2)	4 (1.8)	1.000	0.426
**Laboratory**						
^7^Haematocrit <30%	72 (12.4)	7 (18.4)	52 (17.4)	13 (7.2)	0.875	0.001
^8^White cell count <4×10^9^/L	40 (6.9)	2 (4.5)	19 (6.1)	19 (8.4)	1.000	0.251
^8^White cell count > 15×10^9^/L	11 (1.9)	2 (4.5)	6 (1.9)	3 (1.3)	0.261	0.541
^9^Platelet count <100×10^9^/L	27 (4.6)	2 (5.7)	13 (5.3)	12 (9.0)	1.000	0.165
^10^SGOT >200 IU/L	63 (10.8)	3 (21.4)	43 (32.8)	17 (22.7)	0.549	0.159
^10^SGPT >200 IU/L	44 (7.6)	0 (0)	26 (19.8)	18 (24.0)	0.075	0.286
**Isolate**						
Fully susceptible organism	75 (12.9)	4 (9.1)	30 (9.7)	41 (18.1)	1.000	0.003
MDR	469 (80.7)	39 (88.6)	261 (83.9)	169 (74.8)	0.510	0.004
Reduced susceptibility to FLQ	215 (37.0)	17 (38.6)	127 (40.8)	71 (31.4)	0.781	0.026

^1^Comparison of children < 5 years with children 5–15 years and ^2^ of children < 16 years with adults ≥ 16 years by chi square, Fisher’s exact or Mann–Whitney *U* test as appropriate.

^3^Denominators for age groups are the total for each period.

^4^Denominators for age groups are the total for each site.

^5^median (IQR).

^6^*SBP* – systolic blood pressure.

^7^n = 518.

^8^n = 580.

^9^n = 414.

^10^n = 220.

*FLQ*: Fluoroquinolone.

### Clinical outcomes and the prevalence of severe typhoid

The clinical outcomes of the enrolled typhoid patients are summarised in Table 2 and classified by age range. The prevalence of severe or fatal typhoid at the time of admission in this group of patients was 15.5% (90/581). The most common complications associated with severe disease were gastrointestinal bleeding (43; 7.4%); hepatitis (29; 5.0%); encephalopathy (16; 2.8%); myocarditis (12; 2.1%); pneumonia or pleural effusion (11; 1.9%); intestinal perforation (6; 1.0%); and haemodynamic shock (5; 0.9%). Severe disease was slightly more frequent in adults (39/226; 17.3%) than in children under 16 years (51/355; 14.4%) (Figure 1). The overall case fatality rate was 3/581 (0.5%). All these three fatal cases were adults with combined encephalopathy and haemodynamic shock. The six patients with intestinal perforation, all male, were managed successfully with surgery and antimicrobial therapy. The clinical and laboratory features associated with severe disease are shown in Table 3.

**Table 2:** Outcome in the 581 patients with confirmed typhoid categorised by age group

**Covariate**	**All ages**	**<5 years**	**5**-**15 years**	**≥16 years**	**p value**^**1**^	**p value**^**2**^
	**n = 581**	**n = 44**	**n = 311**	**n = 226**		
Severe or fatal disease	90 (15.5)	4 (9.1)	47 (15.1)	39 (17.3)	0.363	0.348
Gastrointestinal bleeding	43 (7.4)	1 (2.3)	22 (7.1)	20 (8.9)	0.334	0.287
Intestinal perforation	6 (1.0)	0 (0)	2 (0.6)	4 (1.8)	1.000	0.214
Encephalopathy	16 (2.8)	1 (2.3)	7 (2.3)	8 (3.5)	1.000	0.437
Myocarditis	12 (2.1)	0 (0)	9 (2.9)	3 (1.3)	0.609	0.384
Haemodynamic shock	5 (0.9)	0 (0)	0 (0)	5 (2.2)	-	0.009
Renal impairment	4 (0.7)	0 (0)	0 (0)	4 (1.8)	-	0.023
Hepatitis	29 (5.0)	1 (2.3)	18 (5.8)	10 (4.4)	0.488	0.617
Cholecystitis	4 (0.7)	0 (0)	3 (1.0)	1 (0.4)	1.000	1.000
Pneumonia	5 (0.9)	1 (2.3)	2 (0.6)	2 (0.9)	0.328	1.000
Pleural effusion	6 (1.0)	1 (2.3)	3 (1.0)	2 (0.9)	0.412	1.000
Severe anaemia	6 (1.0)	0 (0)	5 (1.6)	1 (0.4)	1.000	0.413
Blood transfusion	3 (0.5)	0 (0)	2 (0.6)	1 (0.4)	1.000	1.000
Death	3 (0.5)	0 (0)	0 (0)	3 (1.3)	-	0.058

^1^Comparison of children < 5 years with children 5–15 years and ^2^ of children < 16 years with adults ≥ 16 years by chi square, Fisher’s exact or Mann–Whitney *U* test as appropriate.

**Table 3:** **Clinical and haematological characteristics of 90 severe and 491 non**-**severe typhoid fever patients**

**Characteristic**	**Severe or fatal n = 90**	**Non**-**severe n = 491**	**OR**	**95% CI**	**p value**
**Signs & symptoms**					
Abdominal pain	45 (50.0)	133 (27.1)	2.69	1.70-4.26	<0.001
Constipation	29 (32.2)	105 (21.4)	1.75	1.07-2.86	0.025
Diarrhoea	62 (68.9)	259 (52.7)	1.98	1.23-3.21	0.005
Vomiting	30 (33.3)	86 (17.5)	2.36	1.43-3.87	0.001
Cough	21 (23.3)	110 (22.4)	1.05	0.62-1.80	0.846
Headache	42 (46.7)	178 (36.3)	1.54	0.98-2.42	0.061
Temperature >40°C	50 (58.1)	268 (56.5)	1.07	0.67-1.70	0.783
Systolic blood pressure <90 mmHg	9 (10.0)	16 (3.3)	3.30	1.41-7.72	0.004
Hepatomegaly	62 (68.9)	241 (49.1)	2.30	1.42-3.71	0.001
Splenomegaly	19 (21.1)	82 (16.7)	1.34	0.76-2.33	0.310
Rash	5 (5.6)	10 (2.0)	2.83	0.94-8.48	0.053
**Haematology**					
Haematocrit <30%^1^	23 (26.7)	49 (11.3)	2.85	1.63-5.01	<0.001
White cell count <4×10^9^/L^2^	10 (11.1)	30 (6.1)	1.92	0.90-4.07	0.086
White cell count >15×10^9^/L^2^	5 (5.6)	6 (1.2)	4.75	1.42-15.90	0.006
Platelet count <100×10^9^L ^3^	7 (9.9)	20 (5.8)	1.77	0.72-4.35	0.211
SGOT > 200 IU/L^4^	33 (63.5)	30 (17.9)	7.99	4.01-15.91	<0.001
SGPT > 200 IU/L^4^	28 (53.8)	16 (9.5)	11.08	5.24-23.47	<0.001

^1^n = 518.

^2^n = 580.

^3^n = 414.

^4^n = 220.

### Risk factors associated with severe typhoid infections

Associations between age, sex, duration of illness prior to admission, hospital site and infection with an organism with an MDR or intermediate ciprofloxacin susceptibility phenotype with severe disease are shown in Table 4. All of these variables were evaluated for associations with severe typhoid through a logistic regression model. After controlling for the effects of confounding, severe disease was independently associated with infection with an organism with intermediate resistance to ciprofloxacin (AOR 1.90; 95% CI 1.18-3.07; p = 0.009) and male sex (AOR 1.04 (1.00-2.57; p = 0.048).

**Table 4:** Factors associated with severe or fatal typhoid fever

**Covariate**	**Severe or fatal n = 90**	**Non severe n = 491**	**OR**	**95% CI**	**p value**	**AOR**	**95% CI**	**p value**
Age (years)^1^	14 (10–23)	12 (7–21)	1.01	0.99-1.03	0.243	1.02	0.99-1.04	0.122
Male	54 (60.6)	242 (49.3)	1.54	0.98-2.44	0.062	1.61	1.00-2.57	0.048
Days ill prior to admission^1^	10 (7–14)	8 (6–10)	1.03	0.99-1.07	0.081	1.04	.99-1.08	0.065
Fully susceptible organism	9 (10.0)	66 (13.4)	0.72	0.34-1.49	0.371	NI		
MDR	78 (86.7)	391 (79.6)	1.66	0.87-3.17	0.123	1.41	0.72-2.75	0.316
Intermediate ciprofloxacin resistance	45 (50.0)	170 (34.6)	1.89	1.20-2.97	0.005	1.90	1.18-3.07	0.009
Site								
Ho Chi Minh City	52/350 (14.9)	298/350 (85.1)	1.00	-	-	1.00	-	-
Dong Thap	38/231 (16.5)	193/231 (83.5)	1.13	0.72-1.78	0.603	1.19	0.72-1.98	0.501

^1^Median (IQR).

*AOR*: adjusted odds ratio; *NI*: Not included.

We hypothesised that the observed association between infection with an organism exhibiting intermediate resistance to ciprofloxacin and disease severity may be related to these individuals having a longer duration of illness before hospital admission, as a result of initially receiving ineffective treatment. However, the median duration of illness prior to hospitalisation for patients infected with an organism with intermediate ciprofloxacin resistance was 8 (IQR: 6–10) days and for patients infected with a ciprofloxacin susceptible organism was also 8 (IQR: 6–13) days (*p* = 0.081).

## Discussion

In this study of 581 Vietnamese patients hospitalised with acute typhoid we identified an independent association of infection with isolates with intermediate susceptibility to ciprofloxacin with severe typhoid. Our research and the research of other groups have previously discussed an impaired response to ofloxacin and ciprofloxacin among patients infected with a ciprofloxacin intermediate isolate [10,11]. A retrospective report in children and adults from New Dehli, India showed an association between severe disease and MDR strains and strains with a decreased susceptibility to fluoroquinolones [16]. In the multivariate analysis in the New Dehli study this relationship with severe disease remained significant for strains exhibiting a decreased susceptibility to fluoroquinolones (OR 3.96, 95% CI 1.39-11.24, p = 0.004). During the period of the study fluoroquinolones, such as ofloxacin and ciprofloxacin, became widely available for treating typhoid infections caused by MDR organisms in outpatients, private clinics and hospitals. Such treatment would be less effective initial treatment for strains with intermediate susceptibility to ciprofloxacin. We anticipated that this might result in a delayed presentation to hospital for patients infected with such strains. In fact there was no significant difference in the duration of illness before admission in these patients compared with those infected with ciprofloxacin susceptible strains.

Our analyses found that severe or fatal disease was slightly more common in adults than children. Previous data regarding the severity of typhoid in children and adults demonstrates variability. Some studies describe typhoid as a severe disease in young children [6,7,13,14], but others characterise it as a comparatively mild, even benign, illness [29-31]. This feature of our findings may lack some generalisability, as severely ill children resident in HCMC may be admitted to one of two specialist paediatric hospitals rather than HTD. Furthermore, typhoid in very young children (< 5 years) may present with unspecific clinical features such as syndromic sepsis, pneumonia, diarrhoea or a viral syndrome and may not be recognised as clinical typhoid [31]. We also found that that severe disease was more common in males and all the cases of intestinal perforation were in males. Previous studies have suggested that disease severity between sexes can vary geographically and has been found to be more common in females [7,15] but also with males, particularly intestinal perforation [4]. It is noteworthy that anaemia was significantly more common among patients with severe disease. We speculate that the reasons for this are multi-factorial including intestinal bleeding, haemolysis and nutritional.

In this analysis severe disease occurred in 15.5% of all typhoid patients and fatal disease in 0.5%. These figures are comparable with studies conducted in Pakistan, Bangladesh, India and the United States [6-8,16]. As only approximately 10% of patients with blood culture confirmed typhoid are hospitalised in this region, these figures likely do not reflect the true frequency of severe disease in the population [32]. The threshold for hospital admission, the availability of antimicrobials in the community without prescription and their use prior to admission will vary between different localities and undoubtedly bias who is admitted to hospital. All the fatal cases from this analysis were adults with encephalopathy and haemodynamic shock; these symptoms have previously been identified as markers of particularly severe disease [33-35].

We found no association of disease severity with an MDR phenotype. In a prospective study of 1,158 children with culture confirmed typhoid fever in Karachi, Bhutta and co-workers found that MDR infections in children in Karachi were associated with higher rates of toxicity, hepatomegaly and hypotensive shock [6]. The 2% mortality in children with an MDR infection in the study from Pakistan was not significantly higher than the 1.4% mortality in those infected with an antimicrobial susceptible strain (OR 1.6, 95% CI; 0.5 - 4.6, p = 0.34). Notably, intermediate susceptibility to fluoroquinolones was not documented in the study from Pakistan. In a previous investigation in Vietnam, which incorporated some data from the first period of this study, higher bacterial counts in the blood were associated with an MDR phenotype [17].

The reason for more severe disease in those infected with strains with intermediate susceptibility to ciprofloxacin may be more complex than merely a poor response to antimicrobial therapy. We speculate that the genotype of the relevant *S*. Typhi organisms may also play an important role in regulating disease severity. Specifically, we suggest an association between bacterial haplotype (assessed by chromosomal single nucleotide polymorphisms (SNPs)) and the pathogenic potential of the strain. There is currently no SNP/disease severity data to support this theory. However, *S*. Typhi isolates originating in Papua New Guinea in the late 1980s and early 1990s exhibited extensive genetic diversity using an insensitive genotyping method (Pulsed field gel electrophoresis (PFGE)), yet a single clone appeared to associated with 11 cases of fatal disease [36]. A further study of 81 *S*. Typhi isolates from 1997–1999, also in Papua New Guinea, showed increased genetic divergence in *S*. Typhi with PFGE, but only a restricted range of PFGE types were found in seven patients with severe or fatal disease [37]. Furthermore, recent studies have shown an association of the ciprofloxacin intermediate phenotype and the IncH1 MDR plasmid with H58 haplotype [38-40]. We have found that this particular haplotype has undergone widespread emergence in the Mekong Delta of Vietnam in the last 10 years [41]. Whether this haplotype is associated with more severe disease requires further investigation.

Our investigation has limitations that need to be considered. Firstly, we only studied hospital admitted cases and our data are, therefore, biased by factors that determine hospital admission such as pre-hospital treatment and access to healthcare. The lack of reliable data concerning pre-hospital antimicrobial therapy is a further drawback. The data are recorded from a period more than ten years ago and, therefore, may not represent the contemporary situation in Vietnam or in other parts of Asia. However, we selected this period as it covered a period of changing antimicrobial susceptibility patterns and strains. In many locations, which are currently endemic for typhoid, antimicrobial resistant strains now dominate the local epidemiology, making it difficult to determine any potential link between resistance and disease severity [41].

## Conclusion

In this large dataset on clinical typhoid and disease severity we found an independent association between infections with strains with intermediate susceptibility to ciprofloxacin and severe typhoid fever. This may reflect the widespread use of fluoroquinolones, particularly ciprofloxacin and ofloxacin, in this area during this period that are not fully effective against these infections. Whether additional host and bacterial factors are implicated in severe disease requires further investigation. Ciprofloxacin and ofloxacin should be used with caution in patients infected with *S*. Typhi that have intermediate susceptibility to ciprofloxacin. Although the newer generation fluorquinolone gatifloxacin is effective for treating such infections it is now unavailable in many countries because of safety concerns. Other alternatives for treating infections with isolates that are MDR and have intermediate susceptibility to ciprofloxacin are extended spectrum cephalosporins and azithromycin [42].

## Competing interests

The authors declare that they have no competing interests.

## Authors’ contribution

CMP, CT, JJF and SB designed the study. CMP, HV, NTC, LTP, VAH, TTH, JW participated in data collection. CMP and CT analysed the data and wrote the first draft. All authors revised the manuscript for important intellectual content and read and approved the final version.

## Pre-publication history

The pre-publication history for this paper can be accessed here:

http://www.biomedcentral.com/1471-2334/14/73/prepub
